# The Influenza Season: A Comparative Study of Admission Rates and Influenza Vaccinations in the Kingdom of Bahrain

**DOI:** 10.7759/cureus.96396

**Published:** 2025-11-08

**Authors:** Mazen M Almulla, Nasser Mansoor, Ali M Haider Ali, Abdulrahman Al-Majmuei, Mohammad Alatoom, Yahya M Waly, Lina Abdelhadi, Leema Alqawasmi

**Affiliations:** 1 Emergency Medicine, Salmaniya Medical Complex, Manama, BHR; 2 Medicine, Royal College of Surgeons in Ireland - Bahrain, Al Muharraq, BHR

**Keywords:** hospital admissions, influenza vaccine, kingdom of bahrain, public health and safety, respiratory infection

## Abstract

Background and objective

Influenza remains a significant global public health challenge, contributing to seasonal rises in healthcare utilization, hospital admissions, and morbidity. Vaccination is recognized as the most effective preventive strategy against influenza infection. This study aimed to examine the association between influenza vaccination status and key clinical outcomes, including hospital admission rates and length of hospital stay, during the 2024-2025 influenza season in the Kingdom of Bahrain.

Methods

A retrospective analysis was conducted involving 5,033 patients who underwent polymerase chain reaction (PCR) testing for influenza in 2024. Patients were categorized into three groups: vaccinated during the current flu season (September 2024 to April 2025), vaccinated in previous seasons, and unvaccinated. Data were extracted from the national I-SEHA electronic health records system. Admission rates, length of hospital stay, and age distribution were analyzed across the groups.

Results

Among the study population, 237 individuals (4.7%) were vaccinated in the current flu season, 1,007 (20%) in prior seasons, and 3,789 (75.3%) remained unvaccinated. Of all hospital admissions, 4.4% were from the currently vaccinated group, 20.3% from those vaccinated in previous seasons, and 75.3% from unvaccinated individuals. The mean length of hospital stay was shortest in the current-season vaccinated group (7.1 days) compared to those vaccinated previously (10.3 days) and the unvaccinated group (9.4 days).

Conclusions

Timely influenza vaccination during the current season was associated with fewer hospital admissions and shorter hospital stays. These findings highlight the importance of seasonal vaccination campaigns and the need for continued public health efforts to promote annual influenza vaccination in order to reduce disease burden and healthcare strain.

## Introduction

Acute respiratory viral infections represent a growing global health concern, accounting for a substantial proportion of emergency department visits. It is estimated that over one-third of such visits are related to respiratory illnesses [1]. These infections are generally classified into upper and lower respiratory tract infections [2]. The upper tract infections include conditions such as rhinitis, pharyngitis, tonsillitis, and laryngitis, which are usually mild and self-limiting, while the lower tract variant includes tracheitis, bronchitis, bronchiolitis, and pneumonia, which are often more severe and can lead to hypoxia and hospitalization [3,4].

Although bacterial pathogens may sometimes be implicated, viral causes remain the most common [5]. Among these, the influenza virus is one of the most significant contributors to seasonal respiratory illness worldwide [5]. In an effort to reduce complications and hospital admissions, public health programmes emphasize the importance of annual influenza vaccination, particularly before the epidemic season, which typically occurs between September and February [2].

In the Kingdom of Bahrain, influenza vaccination is widely accessible through primary healthcare centres, aligning with the Ministry of Health’s initiatives to promote vaccine uptake. This study aims to assess the association between influenza vaccination status and hospital admission rates during the 2024-2025 influenza season in Bahrain. The findings may provide valuable insights into the impact of timely vaccination on reducing hospital burden and improving patient outcomes.

## Materials and methods

This study was approved by the Research Committee of the Governmental Hospitals in the Kingdom of Bahrain (IRB Number: 137-090125) as a retrospective observational review and analysis. This study was conducted in accordance with the Declaration of Helsinki and institutional ethical standards. As it involved anonymised retrospective data, the requirement for informed consent was waived by the Research Committee. Data were collected from both primary healthcare centres and Salmaniya Medical Complex through the national electronic medical records system, I-SEHA, in the Kingdom of Bahrain.

The inclusion criteria consisted of male and female patients of all age groups who underwent testing for suspected influenza infection during the year 2024. The diagnosis was confirmed using the polymerase chain reaction (PCR) method on nasopharyngeal swab samples. Patients were included regardless of whether they were vaccinated during the 2024-2025 influenza season (covering vaccinations administered from September 2024 through April 2025), vaccinated in previous seasons, or unvaccinated.

The following variables were collected: age, gender, vaccination status, date of vaccination, PCR result, and clinical outcome (discharge, hospital admission, or length of hospital stay). Patients with positive PCR results were followed up for their clinical course and outcomes, while those with negative results were excluded from the analysis. The vaccination date was cross-referenced with the PCR test date to ensure that vaccination occurred before infection, thereby confirming valid exposure status.

Any complications observed during testing were documented. Statistical analysis was performed using SPSS Statistics software (IBM Corp., Armonk, NY). Data analysis included descriptive statistics, comparison of admission rates between vaccination groups, and calculation of mean hospital stay durations. Comparisons were conducted across vaccination groups to assess differences in hospital admissions and length of stay. Results were reviewed and verified by an independent data analyst to ensure accuracy and reduce potential bias.

## Results

A total of 5,033 patients were included in the analysis. Patients were categorized into three groups based on their influenza vaccination status: those vaccinated during the current flu season (September 2024 to April 2025), those vaccinated in previous seasons, and those with no documented influenza vaccination. Of the total cohort, 237 patients (4.7%) were vaccinated during the current flu season, 1,007 (20%) had received the vaccine in a prior season, and 3,789 (75.3%) were unvaccinated.

Hospital admission rates were examined across these groups. A total of 2,165 patients (43%) were admitted to the hospital during the study period. Of these admissions, 96 (4.4%) were patients vaccinated during the current flu season, 439 (20.3%) had been vaccinated in a previous season, and 1,630 (75.3%) were unvaccinated. While the within-group admission rates were similar (44.4% for patients vaccinated during the current flu season, 43.6% for those vaccinated in previous seasons, and 43% for unvaccinated individuals), the between-group comparison tells a different story. The total number of admissions was substantially lower in those vaccinated during the current flu season, reflecting a lower overall burden on hospital services from this group. Detailed demographic and clinical figures are presented in Table 1, and laboratory findings and hospital stay data are summarized in Table 2.

**Table 1:** Baseline demographic and clinical characteristics of patients stratified by influenza vaccination status

Characteristic	Category	Vaccinated (September 2024–April 2025)	Vaccinated in previous seasons	No documented influenza vaccination	Total
Mean age, years	Not applicable	40.2	45.4	32.7	35.6
Gender, n (%)	Influenza A	117 (49%)	451 (45%)	2114 (44%)	2682 (47%)
Gender, n (%)	Influenza B	120 (51%)	556 (55%)	1676 (56%)	2352 (54%)
Admission status, n (%)	Admitted	96 (17%)	439 (18%)	1630 (22%)	2165 (21%)
Admission status, n (%)	Not admitted	141 (83%)	568 (82%)	2162 (78%)	2871 (79%)

**Table 2:** Laboratory findings and mean hospital stay duration by influenza vaccination status PCR: polymerase chain reaction

Characteristic	Category	Vaccinated (September 2024–April 2025)	Vaccinated in previous seasons	No documented influenza vaccination	Total
PCR positivity, n	Influenza A	11	65	273	349
PCR Positivity, n	Influenza B	5	12	79	96
Mean hospital stay, days	Not applicable	7.1	10.3	9.4	9.5

The mean length of stay also differed across vaccination groups. Patients vaccinated during the current season had the shortest average hospital stay at 7.1 days. Those vaccinated in previous seasons had the longest stays, with a mean of 10.3 days, while unvaccinated patients had an average stay of 9.4 days. An in-depth analysis is presented in Table 3. These findings suggest a potential association between timely vaccination and reduced hospital resource utilization.

**Table 3:** Summary of hospital stay duration among patients by influenza vaccination status

	Vaccinated (September 2024–April 2025)	Vaccinated in previous seasons	No documented influenza vaccination	Total
N	96	439	1,630	2165
Mean	7.1	10.3	9.4	9.5
Median	6	6	5	5
1st quartile	4	4	3	3
3rd quartile	10	12	10	10
Interquartile range	6	8	7	7
Minimum	1	1	1	1
Maximum	24	331	190	331

**Figure 1:**
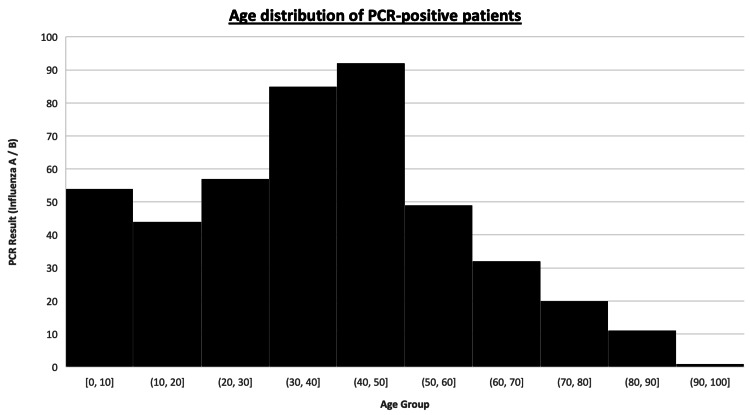
Age distribution of patients who tested positive for influenza A or B by PCR Histogram showing the age distribution of patients who tested positive for influenza A or B by PCR PCR: polymerase chain reaction

The age of admitted patients spanned from infancy to 100 years. The highest number of positive cases was seen in the 40-50-year age group, followed closely by those aged 30-40 years. While PCR-confirmed influenza was observed across all ages, this peak in working-age adults suggests a broad community transmission pattern, not confined to traditionally high-risk groups such as the elderly. See Figure 1 for a clearer representation of this.

## Discussion

This study examined the relationship between influenza vaccination status and hospital outcomes in Bahrain during the 2024-2025 influenza season. The findings suggest that individuals vaccinated during the current flu season were admitted less often and had shorter hospital stays compared to those who were unvaccinated or vaccinated in previous seasons. Although the admission rates within each group appeared similar, the smaller number of hospitalized cases among those vaccinated during the current season indicates a potential protective effect of recent vaccination. These results align with previous research showing that recent influenza vaccination is associated with lower rates of hospitalization and intensive care admission [6,7].

The timing of vaccination appears to play a key role in determining the level of protection. Many studies have assessed vaccine effectiveness in general terms without distinguishing between current and past vaccination. The results of this study show that individuals vaccinated in previous seasons had admission and hospital stay patterns more similar to unvaccinated individuals. This finding reinforces the understanding that immunity from influenza vaccination diminishes over time and highlights the importance of annual vaccination. The influenza vaccine is reformulated annually to match circulating strains, and immunity from earlier doses may provide only partial protection against new variants [8,9]. Previous research has also shown that antibody titres wane significantly within months of vaccination, particularly among older adults [10-12].

A related study by Liu et al. reported a reduction in hospital admissions and mortality over a decade in regions with high vaccine coverage [13]. However, their research focused on long-term coverage trends rather than the effect of vaccination timing. The present study adds to existing knowledge by showing that vaccination during the current season, rather than past immunization, may have a stronger association with improved outcomes. This observation is consistent with findings from Sullivan et al. and Nichol et al., who demonstrated that getting vaccinated every year improves both vaccine performance and overall health outcomes [14,15]. Public health efforts should therefore focus not only on increasing vaccine coverage but also on promoting vaccination early in each season.

The age distribution of patients in this study also provides useful insights. While elderly and very young populations are usually considered at the highest risk for influenza complications, a substantial proportion of hospitalizations occurred among working-age adults. The highest incidence of influenza-positive cases was recorded in the 40-50-year age group, followed by the 30-40-year age group. This pattern suggests that vaccination campaigns should not only target vulnerable populations but also include healthy adults, who represent a large proportion of the workforce and may play a role in community transmission. Similar findings have been reported in studies from Asia and North America, which observed substantial influenza-related morbidity among middle-aged adults [16,17].

This study has several limitations. As a retrospective analysis based on electronic health records, it could not capture individuals with mild illness who did not seek medical attention. Additionally, misclassification bias may have occurred if vaccination records were incomplete, particularly for patients vaccinated outside the national system. Also, focusing only on PCR-confirmed cases may underestimate the total burden of influenza. Potential confounding factors such as age, comorbidities, and healthcare access were not adjusted for in this analysis, which may have influenced the observed associations. Despite these limitations, the consistency of the findings across multiple parameters strengthens their reliability.

The study also has important strengths. It used a large, real-world dataset that included both primary and tertiary care settings. The distinction between current-season, past-season, and unvaccinated groups allowed for more meaningful comparisons. Furthermore, vaccination and clinical outcome data were derived from electronic records rather than self-reported information, which reduces recall bias and improves accuracy.

Overall, the results indicate that current-season influenza vaccination is associated with fewer hospital admissions and shorter hospital stays. These findings underscore the value of timely vaccination as a means of reducing healthcare system burden. Future studies should examine the effect of vaccination timing across different age groups and consider incorporating outpatient and community settings to provide a more comprehensive assessment of vaccine effectiveness.

## Conclusions

This study demonstrates that influenza vaccination during the current flu season is associated with fewer hospital admissions and shorter hospital stays compared to vaccination in previous seasons or no vaccination. While hospitalizations occurred across all age groups, the findings suggest that timely vaccination offers greater protection and reduces the burden on healthcare services. These results underscore the importance of increasing seasonal vaccine uptake and ensuring that immunization efforts are aligned with the timing of peak influenza activity.
